# The role of mucosal IgA in protection against influenza A H1N1 virus infection in a real-world setting

**DOI:** 10.1016/j.ebiom.2026.106359

**Published:** 2026-06-30

**Authors:** Oscar Bladh, Tamás Pongrácz, Katherina Aguilera, Matilda Berkell, Ulrika Marking, Nina Greilert Norin, Ali Rihani, Jessica J. Alm, Florian Krammer, Mikael Åberg, Charlotte Thålin

**Affiliations:** a Department of Clinical Sciences, Danderyd Hospital, Karolinska Institutet, Stockholm, Sweden; b Department of Plant Physiology, National Bioinformatics Infrastructure Sweden (NBIS), Science for Life Laboratory, Umeå University, Umeå, Sweden; c Public Health Agency of Sweden, Solna, Sweden; d Department of Microbiology, Tumor and Cell Biology, and National Pandemic Center, Karolinska Institutet, Solna, Sweden; e Centre for Health Crises, Karolinska Institutet, Solna, Sweden; f Department of Microbiology, Icahn School of Medicine at Mount Sinai, New York, NY, USA; g Center for Vaccine Research and Pandemic Preparedness (C-VaRPP), Icahn School of Medicine at Mount Sinai, New York, NY, USA; h Department of Pathology, Molecular and Cell Based Medicine, Icahn School of Medicine at Mount Sinai, New York, NY, USA; i Ignaz Semmelweis Institute, Interuniversity Institute for Infection Research, Medical University of Vienna, Vienna, Austria; j Ludwig Boltzmann Institute for Science Outreach and Pandemic Preparedness at the Medical University of Vienna, Vienna, Austria; k Department of Medical Sciences, Clinical Chemistry and SciLifeLab Affinity Proteomics, Uppsala University, Uppsala, Sweden

**Keywords:** Influenza A, SARS-CoV-2, Vaccines, Infection, Mucosal antibodies, Correlate of protection

## Abstract

**Background:**

Parenteral vaccines against influenza virus prevent severe disease but provide limited protection against infection, likely due to limited induction of mucosal immunity. However, direct clinical evidence for mucosal antibody-mediated protection against influenza virus infection in humans has been lacking. The aim of this study was to evaluate the role of antigen-specific mucosal IgA as a correlate of protection against influenza virus infection. We investigated H1-specific nasal IgA and serum IgG and the effect on influenza virus infection, with corresponding SARS-CoV-2 data from the same cohort as an internal comparator.

**Methods:**

Using a prospective cohort study design, we analysed paired antigen-specific serum IgG and nasal IgA responses at baseline, after influenza A (H1N1) virus and SARS-CoV-2 infections, and after intramuscular influenza and SARS-CoV-2 vaccination. We then evaluated associations between baseline antibody levels, vaccination, and the risk of subsequent influenza A (H1N1) virus infection or SARS-CoV-2 infections in a 4-month home-based qPCR screening in Stockholm, Sweden (2023–2024). A Poisson regression model with a log offset for time at risk was used to evaluate if baseline antigen-specific nasal IgA or serum IgG levels or recent vaccination was associated with protection against infection. Antibody quantification was performed using a highly sensitive electrochemiluminescence immunoassay (Meso Scale Diagnostics, USA) optimised for mucosal sampling.

**Findings:**

A total of 926 study participants were enrolled. After influenza A (H1N1) virus infection, H1-specific nasal IgA and serum IgG increased (p = 0.007 and p < 0.001 respectively), while vaccination boosted serum IgG (p = 0.006) but not nasal IgA. Baseline nasal H1-specific IgA were lower in H1N1-infected individuals (p = 0.024), and notably, baseline nasal H1-specific IgA above median was associated with reduced H1N1 infection risk (IRR 0.23, 95% CI 0.05–0.75, p = 0.026), while serum IgG and recent vaccination were not (IRR 2.03, 0.70–6.62, p = 0.206 and IRR 1.06, 0.38–3.03, p = 0.917, respectively). Moreover, each two-fold increase in baseline nasal H1-specific IgA levels was associated with a significantly lower infection risk (IRR 0.69, 95% CI 0.51–0.92; p = 0.014). Similar findings were observed for SARS-CoV-2 for mucosal IgA, whereas baseline serum spike-specific IgG above the median was also associated with reduced risk of infection.

**Interpretation:**

Leveraging fit-for-purpose sampling, a validated and highly sensitive assay, and a large real-world cohort, we directly link mucosal IgA to protection against influenza virus infection. While currently employed vaccines provide robust protection against severe disease and hospital admissions, these findings highlight the need for novel approaches generating robust mucosal protection against infection per se, and the need to incorporate mucosal endpoints into next-generation vaccine development aiming for infection-blocking immunity.

**Funding:**

This work was funded by the European Research Council (101164772 - D-MAP), the Swedish Society for Medical Research (SSMF CG-24-0191-B-H-01), the Knut and Alice Wallenberg Foundation (KAW 2024.0108), and the SciLifeLab Pandemic Laboratory Preparedness Program.

**Research in context**

**Evidence before this study**

Respiratory infections remain a major cause of global morbidity and mortality. Although several systemic vaccines reduce disease severity, many do not prevent infection or transmission, likely because they induce limited mucosal immune responses. Despite broad recognition of the importance of mucosal immunity, direct human evidence defining its contribution to protection against natural infection is scarce. The COVID-19 pandemic has provided early indications that mucosal IgA may contribute to reduced SARS-CoV-2 infection, viral replication, and onward transmission, but the available data remain limited. Even less is known about the role of mucosal IgA in protection against natural influenza virus infection. This knowledge gap hampers the rational design of next-generation vaccines and slows both scientific development and investment in mucosal vaccine platforms. To review the current literature, we searched PubMed and medRxiv from database inception to April 2026 using the terms “mucosal” AND (“IgA” OR “immunity”) AND (“Influenza A”) as well as “vaccination” AND (“influenza”) AND “protection against infection”. The search indicated that data on mucosal IgA trajectories post influenza A virus infection and intramuscular influenza vaccination in humans are scarce. Data on mucosal IgA after influenza A virus infection are limited to a few real-world studies with a limited number of study participants, and a handful challenge studies. Similarly, data on mucosal IgA trajectories after intramuscular influenza vaccination are limited to a small number of studies with a limited sample size and a follow up time of up to 5 weeks. Data on associations between mucosal IgA and protection against influenza A infection are largely derived from a small number of controlled human challenge studies, in which protection has been demonstrated in terms of shorter viral shedding, but not against infection per se. While numerous vaccine efficacy studies show that parenteral influenza vaccines protect against disease, none have directly compared antigen-specific mucosal IgA and serum IgG as correlates of protection against symptomatic influenza A infection in a real-world setting. Moreover, no study has systematically evaluated mucosal IgA and serum IgG side-by-side as correlates of protection across both SARS-CoV-2 and influenza A infection.

**Added value of this study**

We followed 926 healthcare workers in a 4-month home-sampling program for qPCR screening of Influenza A (H1N1) and SARS-CoV-2 infection during the 2023–2024 winter season in Stockholm, Sweden. We assessed nasal antigen-specific IgA and serum IgG, together with recent vaccination status, as potential correlates of protection against influenza A (H1N1) or SARS-CoV-2 infection. Additionally, nasal IgA and serum IgG antibodies were measured up to 6 months post infection and vaccination. We found that high pre-existing antigen-specific nasal IgA levels, but not serum IgG levels or recent vaccination status, were significantly associated with protection against infection with both influenza A (H1N1) and SARS-CoV-2. Additionally, antigen-specific nasal IgA increased following natural infection with both viruses, but did not rise after vaccination.

**Implications of all the available evidence**

These findings underscore the importance of antigen-specific mucosal IgA as a correlate of protection against influenza A virus infection and align with emerging evidence of its role in SARS-CoV-2, both viruses with considerable pandemic potential. Strategies aimed at generating robust mucosal IgA responses are urgently needed to reduce infection risk and strengthen pandemic preparedness.

## Introduction

Seasonal influenza virus and SARS-CoV-2 vaccines are effective in preventing symptomatic disease, but their ability to prevent infection is limited,^1^, ^2^, ^3^ likely due to antigenic drift and failure to elicit protective mucosal immunity.^4^, ^5^, ^6^, ^7^ Breakthrough infections remain common in highly vaccinated populations,^1^^,^^8^^,^^9^ causing substantial global illness and loss of life each year.^10^^,^^11^ In addition to placing vulnerable populations at risk for severe disease and death, ongoing viral circulation fuels viral mutations, potentially leading to enhanced transmissibility and/or increased virulence.^12^ In the case of influenza viruses, the circulation of multiple influenza viral strains in both mammals and birds, combined with the enormous genetic diversity of the virus, increases the risk of genetic reassortment and, ultimately, antigenic shift, events that may trigger a future pandemic.^13^

Mucosal IgA in the respiratory tract plays a critical role in host defence by neutralising pathogens at the point of viral entry and preventing their adherence to the epithelium,^14^, ^15^, ^16^ thereby limiting respiratory viral infections and transmission. The mechanisms by which IgA in the respiratory mucosa protects against infection are multifactorial. IgA in the respiratory mucosa exists primarily in a polymeric form, consisting of two or more monomeric IgA molecules linked by the joining chain (JC).^14^ Epithelial cells in the mucosa express the polymeric Ig receptor (pIgR), which facilitates the transcytosis of locally synthesised, JC-containing polymeric IgA across the epithelium into external secretions. pIgR is proteolytically cleaved during transcytosis to release its extracellular domain bound to polymeric IgA as the secretory component (SC), ultimately giving rise to secretory IgA. Consequently, secretory IgA is a complex of polymeric IgA, JC, and SC. This polymeric structure protects secretory IgA from proteolytic degradation,^17^ broadens its cross-binding capacity,^18^^,^^19^ and increases its avidity. Taken together, these combined structural and functional attributes render secretory IgA well suited for protection at mucosal surfaces.

While systemic immune responses to influenza virus infection are well characterised, surprisingly few clinical studies have examined mucosal IgA responses following natural influenza infection in humans.^13^ In contrast, several recent reports have shown that SARS-CoV-2 infections induce robust mucosal IgA responses.^7^^,^^20^, ^21^, ^22^, ^23^, ^24^, ^25^, ^26^ Intramuscularly delivered influenza virus- and SARS-CoV-2 vaccines, however, induce limited mucosal IgA responses,^5^^,^^6^^,^^20^^,^^21^^,^^27^ which likely explains their inability to prevent infection and onward transmission. Investigations into correlates of protection against COVID-19 and influenza have predominantly focused on circulating antibodies, particularly those of the IgG isotype, and the protective role of mucosal IgA is not as well characterised. Investigating correlates of protection against SARS-CoV-2 and influenza virus infection *per se* is furthermore challenging because many infections, particularly asymptomatic or mild cases, go unnoticed without systematic and frequent screening. We have overcome this challenge by implementing regular quantitative polymerase chain reaction (qPCR) screening programs following serum and mucosal sampling of healthcare workers in the longitudinal COMMUNITY cohort (NCT06784739), enabling the detection of also mild infections. Using this approach, we have repeatedly shown that high levels of spike-specific mucosal IgA are associated with protection against infection with various SARS-CoV-2 variants.^7^^,^^23^^,^^25^ Although reports have linked nasal anti-haemagglutinin (HA) and anti-neuraminidase (NA) IgA titres to reduced influenza virus infection-associated illness and accelerated viral clearance,^28^^,^^29^ investigations of mucosal correlates of protection against influenza virus infection in humans are limited to challenge studies.^5^^,^^29^

Here, we address key knowledge gaps regarding mucosal immune responses to natural influenza A virus infections and their role in protection against infection. We characterise anti-H1 serum IgG and mucosal IgA responses following influenza A H1N1 virus infection and seasonal influenza virus vaccination and examine their associations with the risk of subsequent influenza A H1N1 virus infection in over 900 healthcare workers from the COMMUNITY cohort. For context, we also include corresponding SARS-CoV-2 data from the same cohort as an internal comparator.

## Methods

### Study design

In this prospective cohort study, participants were recruited via the ongoing COMMUNITY study (NCT06784739) in Stockholm, Sweden, which longitudinally explores immune responses to respiratory infections and vaccinations through the collection of blood, nasal secretions and saliva every four months since enrolment in April 2020. Baseline nasal secretion and serum samples collected in December 2023 were used for this sub-study, and data on vaccinations was obtained from Swedish national registers. Associations between baseline antibody titres and vaccination status and subsequent influenza A H1N1 virus or SARS-CoV-2 infections were determined through a 4-month well-established home-sampling program for qPCR screening during the winter season of 2023–2024 (Figure S1). At symptom onset, self-administered nasal/oropharyngeal/saliva swabs were sent in pre-stamped envelopes to the National Pandemic Center (NPC) at Karolinska Institutet, Stockholm, for reverse transcription qPCR within 48 h from sampling using previously described home-based sampling kits and protocols.^25^

### Ethics

All study participants provided written informed consent, and the study protocol was approved by the Swedish Ethical Review Authority (dnr 2020-01653). The study was conducted in accordance with the ethical principles of the 2024 Declaration of Helsinki.

### Nasal secretion collection

The soft end of a FLOQSwab (cat nr. CP501CS01, COPAN Diagnostics Inc., United States) was inserted and twirled for 5 s in the outer nostril, then placed in a 15 mL Falcon tube with 1 mL of Dulbecco's phosphate buffer solution 1×. The solution was aliquoted after the tubes had been vortexed for 10 s. All sampling was performed by trained study personnel. To correct for factors such as nasal sampling efficacy, stress and circadian rhythm that may affect the levels of secreted IgA, ratios between antigen-specific IgA and total IgA concentration in the same nasal secretion sample were calculated. If the total IgA concentration was below the limit of detection (LoD), the sample was excluded, regardless of antigen-specific IgA above LoD.

### Serum collection

Venipuncture was used to collect blood samples and blood was transferred into serum separator tubes (SST II, BD Vacutainer®). Samples were left to clot for a minimum of 30 min at room temperature before being centrifuged at 2000 g for 10 min. Subsequently, the supernatant was aliquoted and frozen at −80 °C within 1–2 h for later analyses.

### Antibody quantification

We have previously shown that antigen-specific IgA and secretory IgA levels in nasal secretions have a strong correlation.^30^ In this study we consequently analysed nasal antigen-specific IgA as a proxy for antigen-specific secretory IgA. Antigen-specific IgA and IgG levels were quantified in nasal secretions (dilution 1:1000) and serum (dilution 1:50,000), respectively. Specific antigen targets were influenza A H1N1 (Influenza A haemagglutinin protein from A/Wisconsin/67/2022 (H1N1); T4 fibritin trimerisation motif, TEV protease site) and wild-type spike protein for SARS-CoV-2 (soluble ectodomain with T4 trimerisation domain; C-terminal Strep-Tag and His-Tag). Both antigens were generated and purified by Meso Scale Diagnostics (MSD, Maryland, USA). The Influenza A H1N1 assay was part of a prototype COVID-Flu-RSV panel produced and qualified by MSD and the SARS-CoV-2 WT assay was part of the SARS-CoV-2 panel 31 (catalogue number IgG: K15642U and IgA: K15644U) and analysed according to the manufacturer's instructions. Reference Standard 1 (catalogue number C00ADK-2) was provided with the kits and is a serum-based calibrator quality-controlled by MSD and was used for standard curve generation. For the 7-point standard curve, a 4-fold serial dilution was used with two replicates at each calibrator level and an additional zero calibrator blank. Detection was performed using SULFO-TAG conjugated mouse monoclonal anti-human IgG (catalogue number D21ADF) or anti-human IgA (catalogue number D21ADE) antibodies supplied with the kits. Antibody concentrations were interpolated from the respective standard curves and by correcting for dilution, the final antibody concentrations in undiluted samples were obtained. Total IgA concentrations were quantified using the Isotyping Panel 1 Human/NHP Kit (MSD, catalogue number K15203D) following the manufacturer's instructions (dilution 1:5000). The ratios between antigen-specific IgA and total IgA concentration were multiplied by 1 × 10^7^ for graphical purposes. If H1-specific nasal IgA values were below the LoD, but total nasal IgA values were detectable, the normalised nasal IgA values were set to 1e-1. If normalised spike-specific nasal IgA or spike-specific serum IgG values were below cut-off, the values were set to cut-off/√2 for graphical purposes. Cut-off value for spike-specific IgG in serum was provided by the manufacturer (1960 arbitrary units (AU)/mL), whereas the cut-off-value for normalised nasal spike-specific IgA was set at 3.6 AU/mL, by using the mean + 3SD of fourteen pre-pandemic nasal secretion samples. Results from the antibody assays are expressed in AU/mL.

### qPCR diagnostics

RNA extraction and RT-qPCR analyses were performed using automated magnetic bead–based extraction and in-house one-step multiplex assays targeting influenza A (pan-INFA and H1N1) and human RNase P, as well as the Sansure IVD kit for SARS-CoV-2 detection. Detailed protocols are provided in the Supplementary Methods.

### Statistical analyses

Poisson regression models were used to explore the risk of viral infection during follow-up, with a log offset for time at risk. Time at risk was defined as the number of weeks from baseline in the screening study to the earliest of: (1) a positive qPCR result for a virus within the same viral family, (2) vaccination with an antigen from the same viral family, or (3) the end of follow-up (March 10, 2024), set to correspond well to the biologically relevant exposure window based on national epidemiological data on SARS-CoV-2 and Influenza A circulation.^31^ In models evaluating correlates of protection, age, sex (where applicable), mucosal IgA, and serum IgG levels were included as covariates. Sex was omitted from influenza A H1N1 models because only one infected participant was male, precluding stable estimation of sex as a covariate. Participants with an infection or vaccination event on the day of baseline sampling, and those lacking a mucosal sample at baseline, were excluded. The reduced risk of infection among participants with nasal antigen-specific antibody levels above the median, as well as the stronger protection associated with each doubling of nasal antibody levels, were assessed in separate models. Only participants with nasal IgA levels above the assay cut-off were included in the latter analyses. In separate models assessing vaccine effectiveness, time at risk was defined to start 14 days after vaccination, or from baseline for non-vaccinated individuals. Participants vaccinated 31–180 days prior to baseline, as well as those who were infected within 14 days following vaccination, were excluded from the analyses. These models did not include serum IgG or mucosal IgA levels as covariates. Antibody levels in unpaired groups were compared using the Mann–Whitney U test, whereas paired antibody levels were compared using the Wilcoxon matched-pairs signed-rank test. Categorical variables are presented as counts and percentages. Continuous variables are presented as median (IQR), except for age, which is presented as median (min-max). Poisson regression analyses were performed using R version 4.3.2. Within R, data manipulation was facilitated by a suite of packages including tidyverse, lubridate, among others. Data visualisation was performed using Graphpad Version 10.0.3.

### Role of funders

The funders had no role in the study design, data collection, data analysis, interpretation of results, or writing of this report. The decision to submit the manuscript for publication was made solely by the authors.

## Results

In December 2023, 1049 participants were sampled for SARS-CoV-2 spike-specific and influenza A H1-specific nasal IgA and serum IgG levels as part of a regular follow-up within the ongoing longitudinal COMMUNITY healthcare worker cohort (NCT06784739). Of these, 926 participants were enrolled in a 4-month qPCR screening program. None of the PCR-confirmed infections identified during the screening period resulted in hospital admission. In addition, we assessed antigen-specific responses in nasal secretions (IgA) and serum (IgG) 4–6 months after infection with either influenza A H1N1 or SARS-CoV-2 and 1–3 months after vaccination against influenza A with a seasonal tetravalent inactivated split-virion influenza vaccine or SARS-CoV-2 with an mRNA vaccine.

### Influenza A H1N1 virus and SARS-CoV-2 infections boost IgG in blood and IgA in the respiratory mucosa

**Fig. 1:**
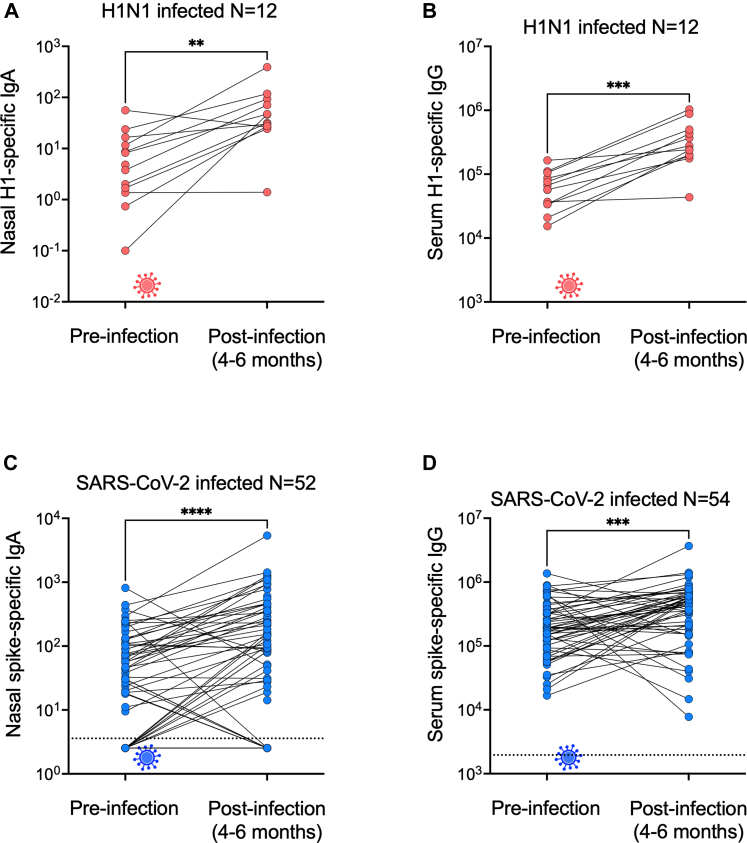
**Antigen-specific nasal IgA and serum IgG following influenza A H1N1 virus and SARS-CoV-2 infection.** A) H1-specific nasal IgA prior to and 4–6 months post influenza A H1N1 virus infection, B) H1-specific serum IgG prior to and 4–6 months post influenza A H1N1 virus infection, C) spike-specific nasal IgA prior to and 4–6 months post SARS-CoV-2 infection, and D) spike-specific serum IgG prior to and 4–6 months post SARS-CoV-2 infection. Antigen-specific nasal IgA levels were normalised to total nasal IgA levels in the same sample and are expressed in AU/ml. Black dotted line represents cut-off level for spike-specific IgA and IgG (not available for H1-specific IgA and IgG). Statistical comparisons were performed using the Wilcoxon matched-pairs signed rank test. ∗∗p < 0.01; ∗∗∗p < 0.001; ∗∗∗∗p < 0.0001.

Participants who had an available follow-up sample and tested qPCR-positive for influenza A H1N1 virus (n = 12) or SARS-CoV-2 infection (n = 54) during the PCR-screening were included in the post infection analysis of mucosal IgA and serum IgG levels (Table 1). Antigen-specific nasal IgA levels increased significantly 4–6 months post both influenza A H1N1 virus infections (median fold change 8.0, p = 0.007) and SARS-CoV-2 infections (median fold change 3.0, p < 0.001) (Fig. 1A, C). Antigen-specific serum IgG levels also increased after both H1N1 virus (median fold change 4.5, p < 0.001) and SARS-CoV-2 infections (median fold change 2.5, p < 0.001) (Fig. 1B, D).

**Table 1:** Clinical characteristics of participants with qPCR-confirmed influenza A (H1N1) or SARS-CoV-2 infection included in longitudinal antibody analyses.

	Overall (N = 66)	H1N1 (N = 12)	SARS-CoV-2 (N = 54)
**Age**			
Median [Min, Max]	52 [29, 70]	55 [40, 66]	51 [29, 70]
**Sex**			
Female	56 (85%)	11 (92%)	45 (83%)
Male	10 (15%)	1 (8%)	9 (17%)
**Days from baseline sample to infection**			
Median [Q1, Q3]	13 [7, 23]	22 [12, 31]	17 [7, 30]
**Days from infection to follow-up sample**			
Median [Q1, Q3]	152 [140, 157]	138 [127, 154]	143 [129, 155]

Q = quartile.

### SARS-CoV-2 and influenza virus vaccinations boost IgG in blood but not IgA in the respiratory mucosa

**Fig. 2:**
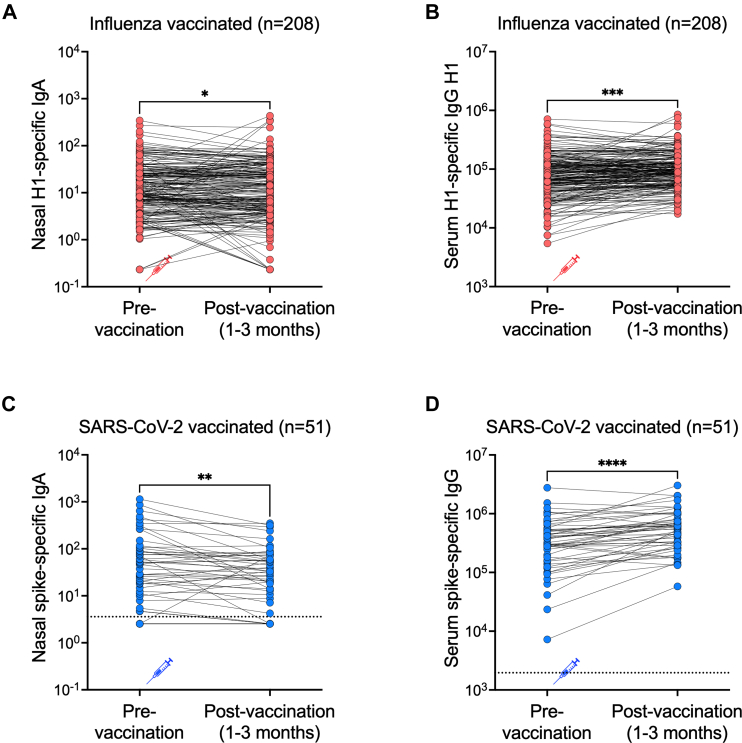
**Antigen-specific nasal IgA and serum IgG following influenza virus and SARS-CoV-2 vaccination.** A) H1-specific nasal IgA prior to and 1–3 months post influenza virus vaccination, B) H1-specific serum IgG prior to and 1–3 months post influenza virus vaccination, C) spike-specific nasal IgA prior to and 1–3 months post SARS-CoV-2 vaccination, and D) spike-specific serum IgG prior to and 1–3 months post SARS-CoV-2 vaccination. Antigen-specific nasal IgA levels were normalised to total nasal IgA levels in the same sample and are expressed in AU/ml. Black dotted line represents cut-off level for spike-specific IgA and IgG (not available for H1-specific IgA and IgG). Statistical comparisons were performed using the Wilcoxon matched-pairs signed rank test. ns = Non-significant, ∗p < 0.05; ∗∗p < 0.01; ∗∗∗p < 0.001; ∗∗∗∗p < 0.0001.

Nasal secretions and blood samples were collected from participants before and 1–3 months after receipt of a seasonal influenza or a SARS-CoV-2 vaccine during the preceding winter season of 2022–2023. Participants with a PCR-confirmed infection with the corresponding virus during follow-up were excluded (influenza A, n = 6; SARS-CoV-2, n = 5), leaving 51 individuals in the SARS-CoV-2 vaccination group and 208 in the influenza vaccination group (Table 2). While antigen-specific serum IgG levels increased after both vaccinations (p < 0.001), a slight decrease in antigen-specific nasal IgA levels was observed after both influenza vaccination and SARS-CoV-2 vaccination (p = 0.015 and p = 0.006, respectively) (Fig. 2A–D).

**Table 2:** Clinical characteristics of participants receiving seasonal influenza or SARS-CoV-2 vaccination included in longitudinal antibody analyses.

	Overall (N = 259)	Influenza (N = 208)	SARS-CoV-2 (N = 51)
**Age**			
Median [Min, Max]	53 [22.0, 69.0]	53 [22, 69]	53 [34, 69]
**Sex**			
Female	238 (92%)	190 (91%)	48 (94%)
Male	21 (8%)	18 (9%)	3 (6%)
**Days from baseline sample to vaccination**			
Median [Q1, Q3]	50 [43, 56]	50 [46, 56]	40 [27, 65]
**Days from vaccination to follow-up sample**			
Median [Q1, Q3]	82 [75, 88]	81 [76, 85]	91 [63, 105]

Q = quartile.

### Antigen-specific IgA in the respiratory mucosa was associated with protection against influenza A H1N1 virus and SARS-CoV-2 infection

Next, we explored associations between antigen-specific IgA in nasal secretions and IgG in blood and the risk of influenza A H1N1 virus and SARS-CoV-2 infections. After exclusion of those with a same-day infection or vaccination at baseline or missing baseline data, 883 participants were included in the influenza A H1N1 (Table 3) and 842 in the SARS-CoV-2 (Table 4) correlate-of-protection analyses.

**Table 3:** Baseline characteristics of participants included in the influenza antibody correlate of protection analysis.

	Overall (N = 883^a^)	H1N1 (N = 14)	No infection (N = 869)
**Age**			
Median [Min, Max]	53 [19, 76]	56 [44, 66]	53 [19, 76]
**Sex**			
Female	775 (88%)	13 (93%)	762 (88%)
Male	108 (12%)	1 (7%)	107 (12%)
**Vaccine during study follow-up**			
Yes	45 (5%)	1 (13%)	44 (5%)
No	838 (95%)	13 (87%)	825 (95%)
**Vaccine 1 month before baseline sampling**			
Yes	408 (46%)	7 (50%)	401 (46%)
No	475 (54%)	7 (50%)	468 (54%)

a Of the 883 participants included, 852 had detectable baseline H1-specific nasal IgA and were included in the log2-transformed Poisson model; all 13 H1N1 infections in this subset occurred in female participants.

**Table 4:** Baseline characteristics of participants included in the SARS-CoV-2 antibody correlate of protection analysis.

	Overall (N = 842^a^)	SARS-CoV-2 (N = 55)	No infection (N = 787)
**Age**			
Median [Min, Max]	53 [19, 76]	50 [29, 70]	53 [19, 76]
**Sex**			
Female	739 (88%)	46 (84%)	693 (88%)
Male	103 (12%)	9 (16%)	94 (12%)
**Vaccine during study follow-up**			
Yes	37 (4%)	1 (2%)	36 (5%)
No	805 (96%)	54 (98%)	751 (95%)
**Vaccine 1 month before baseline sampling**			
Yes	104 (12%)	6 (11%)	98 (13%)
No	738 (88%)	49 (89%)	689 (87%)

a Of the 842 participants included, 834 had detectable baseline spike-specific nasal IgA and were included in the log2-transformed Poisson model, in which 53 SARS-CoV-2 infections occurred.

**Fig. 3:**
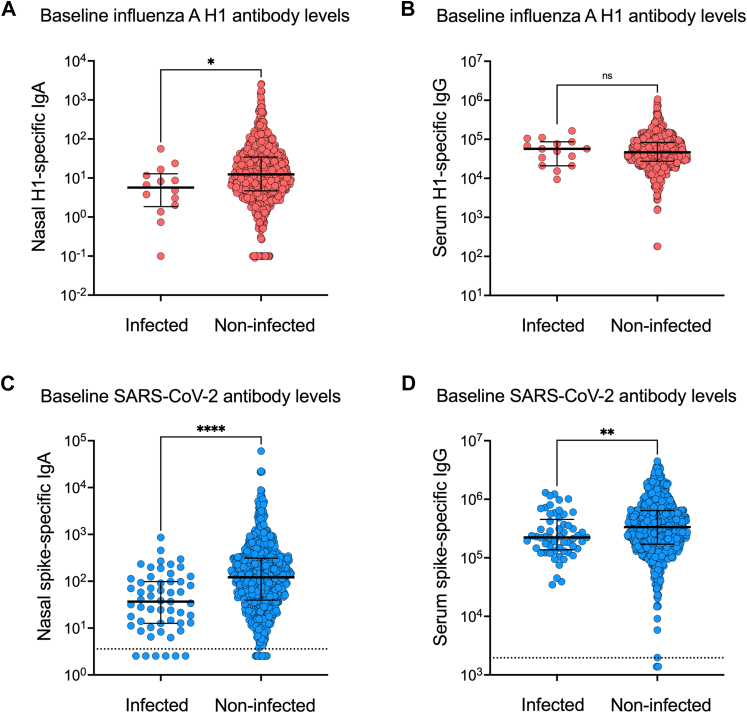
**Associations between baseline antibody levels and subsequent infection.** Baseline antigen-specific nasal IgA and antigen-specific serum IgG levels in individuals infected and non-infected with influenza A (A–B) and SARS-CoV-2 (C–D). Horizontal bars indicate the median and interquartile range. Nasal antigen-specific IgA levels were normalised to total nasal IgA levels in the same sample and are expressed in AU/ml. Black dotted line represents cut-off level for spike-specific IgA and IgG (not available for H1-specific IgA and IgG). Statistical comparisons were performed using the Mann–Whitney U test. ns = non-significant; ∗p < 0.05; ∗∗p < 0.01; ∗∗∗∗p < 0.0001.

**Fig. 4:**
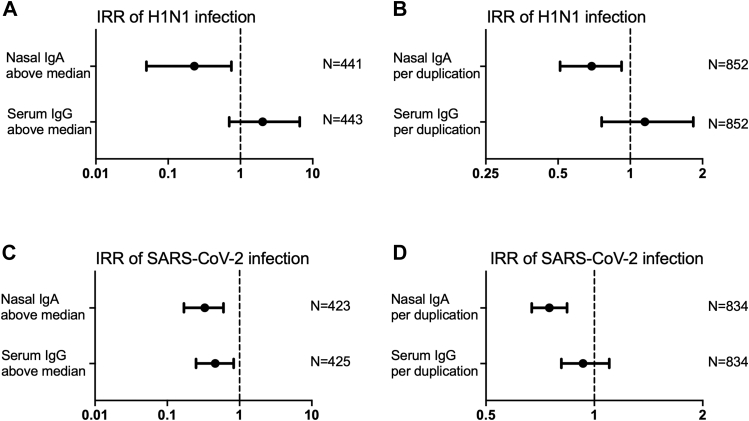
**Associations between antigen-specific nasal IgA and serum IgG levels and the risk of subsequent influenza A H1N1 virus or SARS-CoV-2 infection.** A) IRR of influenza A H1N1 virus infection among participants with H1-specific nasal IgA or serum IgG levels above median at baseline as compared with participants with lower levels (≤median), B) IRR of influenza A H1N1 virus infection per duplication of nasal H1-specific IgA and serum IgG levels in participants with detectable H1-specific antibody levels, C) IRR of SARS-CoV-2 infection among participants with spike-specific nasal IgA or serum IgG above median at baseline as compared with participants with lower levels (≤median), and D) IRR of SARS-CoV-2 infection per duplication of nasal SARS-CoV-2 spike-specific IgA and serum IgG levels in participants with detectable spike specific antibody levels. Antigen-specific nasal IgA levels were normalised to total nasal IgA levels in the same sample. IRR was determined by Poisson regression models with log(time at risk) as offset. Models for panels A and B were adjusted for age, nasal IgA and serum IgG; sex was omitted from influenza A H1N1 models because only one infected participant was male, precluding stable estimation of sex as a covariate. Models for panels C and D (SARS-CoV-2) were additionally adjusted for sex. IRR; incidence rate ratio. N = number of participants in regression analysis.

Median baseline nasal antigen-specific IgA levels were lower in individuals who later became infected with influenza A H1N1 virus (p = 0.024) or SARS-CoV-2 (p < 0.001). However, antigen-specific serum IgG levels did not differ between individuals who later became infected with influenza A H1N1 and those who remained uninfected (p = 0.893) but were slightly lower in those with subsequent SARS-CoV-2 infection (p = 0.009) (Fig. 3A–D). No meaningful correlations were observed between age or sex and baseline antigen-specific nasal IgA or serum IgG levels (R values ranged from −0.051 to 0.087), except for a weak but statistically significant correlation between age and serum spike-specific IgG (R = 0.276, p < 0.001). Participants with H1-specific nasal IgA levels above the median at baseline (n = 441) had a significantly reduced risk of subsequently testing positive for influenza A H1N1 virus infection during the screening period with an incidence rate ratio (IRR) of 0.23 (CI: 0.05–0.75, p = 0.026) (Fig. 4A). In participants with detectable H1-specific nasal IgA levels at baseline (n = 852), the risk of influenza A H1N1 virus infection was also reduced with every duplication of baseline H1-specific nasal IgA level (IRR: 0.69, CI: 0.51–0.92, p = 0.014) (Fig. 4B). H1-specific serum IgG levels above the median were not associated with a reduced risk of influenza A H1N1 virus infection (IRR: 2.03, CI: 0.70–6.62, p = 0.206), nor was a duplication of the baseline H1-specific serum IgG level (IRR: 1.15, CI: 0.76–1.83, p = 0.556) (Fig. 4A–B). Similarly, participants with spike-specific nasal IgA levels above the median at baseline (n = 423) had a reduced risk of testing positive for SARS-CoV-2 infection (IRR: 0.33, CI: 0.17–0.60, p < 0.001) (Fig. 4C), and every duplication of baseline spike-specific nasal IgA level, in participants with detectable spike-specific nasal IgA level at baseline (n = 834), was associated with a reduced infection risk (IRR: 0.75, CI: 0.67–0.84, p < 0.001) (Fig. 4D). Baseline spike-specific serum IgG above the median was associated with a reduced risk of testing positive for SARS-CoV-2 infection (IRR: 0.46, CI: 0.25–0.83, p = 0.012), but no reduction was observed per duplication of baseline level (IRR: 0.93, CI: 0.81–1.10, p = 0.342) (Fig. 4C–D).

Although vaccines remain highly effective at preventing severe disease, there was no association between vaccination and the risk of mild influenza A H1N1 virus or SARS-CoV-2 infection during the follow-up.

Finally, considering the lack of vaccine-induced IgA in the respiratory mucosa, we investigated the association between seasonal tetravalent influenza virus vaccination, SARS-CoV-2 mRNA vaccination and protection against infection with corresponding virus. The 926 participants enrolled in the qPCR screening program were stratified based on whether they had been vaccinated within 30 days prior to baseline sampling. Poisson regression models were used to compare participants vaccinated within 30 days before baseline sampling with those not vaccinated during the preceding 6 months. Participants vaccinated 31–180 days before baseline were excluded (influenza: n = 17; SARS-CoV-2: n = 62), leaving 895 participants for the influenza A H1N1 analysis and 809 for the SARS-CoV-2 analysis; of whom 419 and 109, respectively, had been vaccinated within 30 days before baseline (Tables 5 and 6).

**Table 5:** Baseline characteristics of participants included in the influenza vaccine effectiveness analysis.

	Overall (N = 895)	H1N1 (N = 15)	No infection (N = 880)
**Age**			
Median [Min, Max]	53 [19, 76]	56 [44, 68]	53 [19, 76]
**Sex**			
Female	786 (88%)	14 (93%)	772 (88%)
Male	109 (12%)	1 (7%)	108 (12%)
**Vaccine during study follow-up**			
Yes	46 (5%)	2 (13%)	44 (5%)
No	849 (95%)	13 (87%)	836 (95%)
**Vaccine 1 month before baseline sampling**			
Yes	419 (47%)	8 (53%)	411 (47%)
**No vaccine within 6 months of baseline sampling**^a^			
Yes	476 (53%)	7 (47%)	469 (53%)

Vaccine refers to seasonal influenza vaccination.

a Participants in the no vaccine group had not received influenza vaccination within 180 days before baseline. Participants vaccinated 31–180 days before baseline were excluded.

**Table 6:** Baseline characteristics of participants included in the SARS-CoV-2 vaccine effectiveness analysis.

	Overall (N = 809)	SARS-CoV-2 (N = 52)	No infection (N = 757)
**Age**			
Median [Min, Max]	52 [19, 76]	50 [30, 70]	52 [19, 76]
**Sex**			
Female	711 (88%)	43 (83%)	668 (88%)
Male	98 (12%)	9 (17%)	89 (12%)
**Vaccine during study follow-up**			
Yes	39 (5%)	1 (2%)	38 (5%)
No	770 (95%)	51 (98%)	719 (95%)
**Vaccine 1 month before baseline sampling**			
Yes	109 (13%)	6 (11%)	103 (14%)
**No vaccine within 6 months of baseline sampling**^a^			
Yes	700 (87%)	46 (89%)	654 (86%)

Vaccine refers to seasonal SARS-CoV-2 vaccination.

a Participants in the no vaccine group had not received SARS-CoV-2 vaccination within 180 days before baseline. Participants vaccinated 31–180 days before baseline were excluded.

**Fig. 5:**
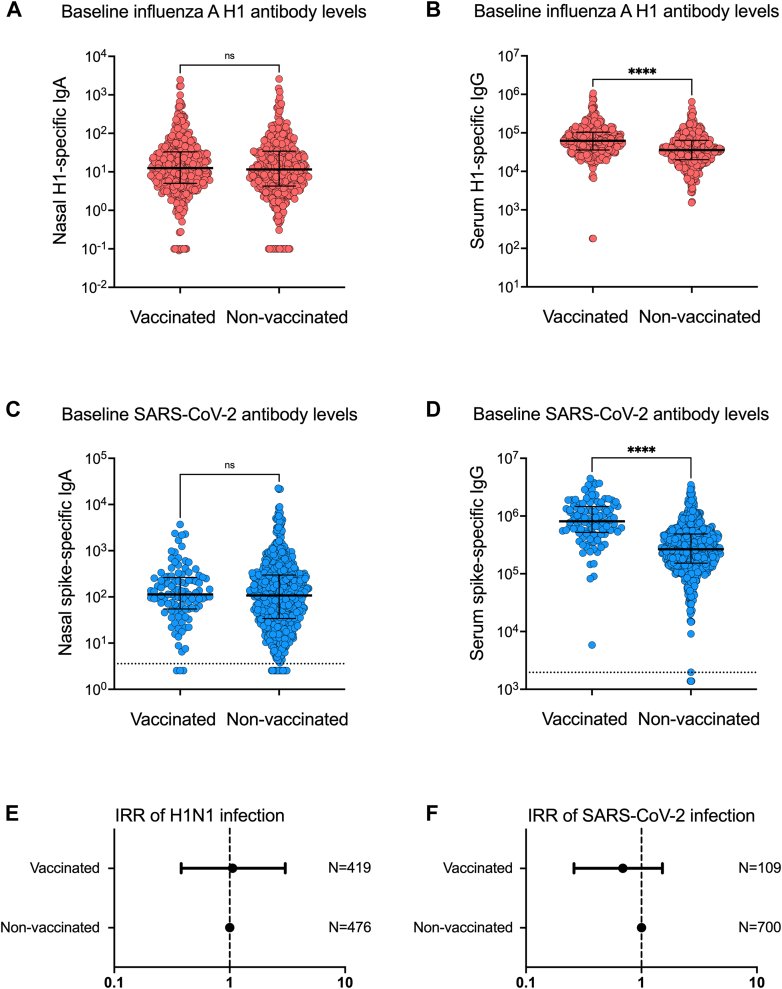
**Associations between the seasonal influenza virus vaccine, SARS-CoV-2 mRNA vaccine and protection against infection with corresponding virus.** Baseline antigen-specific nasal IgA and antigen-specific serum IgG levels in individuals vaccinated and non-vaccinated against influenza (A–B) and SARS-CoV-2 (C–D). Horizontal bars indicate the median and interquartile range. E) IRR of influenza A H1N1 virus infection among participants who received the seasonal tetravalent influenza virus vaccine within one month prior to baseline sampling compared to those who had not received the vaccine in the six months preceding baseline sampling and F) IRR of SARS-CoV-2 infection among participants who received a SARS-CoV-2 mRNA vaccine within one month prior to baseline sampling compared to those who had not received a vaccine in the six months preceding baseline sampling. Antigen-specific nasal IgA levels were normalised to total nasal IgA levels in the same sample and are expressed in AU/ml. Black dotted line represents cut-off level for spike-specific IgA and IgG (not available for H1-specific IgA and IgG). Statistical comparisons were performed using the Mann–Whitney U test. ns = non-significant; ∗∗∗∗p < 0.0001. IRR was determined by a Poisson regression model adjusting for age and sex. IRR = incidence rate ratio. N = number of participants in regression analysis.

Nasal antigen-specific IgA levels did not differ between vaccinated and unvaccinated individuals (p = 0.175 for influenza A H1N1; p = 0.390 for SARS-CoV-2). In contrast, and as expected, antigen-specific serum IgG levels were significantly higher in vaccinated compared to unvaccinated individuals for both influenza A H1N1 and SARS-CoV-2 (p < 0.001 for both comparisons) (Fig. 5A–D). There was no association between recent influenza virus or SARS-CoV-2 vaccination and risk of influenza A H1N1 virus or SARS-CoV-2 infection (IRR: 1.06, CI: 0.38–3.03, p = 0.917 and IRR: 0.69, CI: 0.26–1.52, p = 0.398, respectively) (Fig. 5E–F). Full results from the Poisson regression models, including both unadjusted and adjusted IRRs, are presented in Table S1.

## Discussion

Our findings show that influenza A H1N1 virus infections elicit robust antibody responses in both blood and the respiratory mucosa and support previous reports demonstrating a similar pattern post SARS-CoV-2 infections. We furthermore demonstrate an association between high levels of anti-H1 IgA in the respiratory mucosa and a reduced risk of influenza A H1N1 virus infection, while we also corroborate our recent data showing that high spike-specific mucosal IgA levels correlate to a substantial reduction in the risk of SARS-CoV-2 infection. In line with prior data, intramuscular vaccination did not induce or boost IgA in the respiratory mucosa. While these vaccines are highly effective at preventing symptomatic and severe disease, which is their primary objective, they were not associated with protection against infection per se with the corresponding respiratory viruses.

Numerous studies have shown that intranasal administration of live attenuated influenza vaccine (LAIV) induces durable mucosal IgA responses.^27^^,^^32^, ^33^, ^34^ Real-world clinical data on mucosal immune responses to infection in humans are however, limited. Influenza A virus infection elicited detectable influenza virus-specific secretory IgA in nasal washes for up to 300 days in one small study,^35^ and two challenge studies have shown transient induction of nasal IgA after influenza A H1N1 virus exposure, with a follow-up period of up to 8 weeks.^5^^,^^29^ Here, we extend these findings by showing that H1-specific IgA antibodies remain elevated in nasal secretions for up to six months following natural influenza A H1N1 virus infection. Several reports have demonstrated mucosal IgA responses following SARS-CoV-2 infection, with antigen-specific IgA remaining detectable in the respiratory mucosa for up to two years post infection.^36^ Our data corroborate these findings, showing that spike-specific nasal IgA remains elevated for at least six months following recent SARS-CoV-2 variant infections.

Although nasal IgA has repeatedly been shown to protect against influenza virus infection in animal models,^37^, ^38^, ^39^ human data are largely derived from controlled challenge studies. Gould et al. demonstrated that higher baseline nasal IgA levels were associated with shorter viral shedding following influenza A H1N1 challenge.^29^ Another challenge study performed by Bean et al. found that individuals who experienced prolonged viral shedding had lower systemic and mucosal influenza-specific antibody levels.^5^ In this study we demonstrate that antigen-specific nasal IgA is associated with protection against influenza A H1N1 virus infection in a real-world prospective cohort setting, providing direct human evidence beyond prior controlled challenge studies. In contrast, H1-specific serum IgG was not associated with protection against influenza A H1N1 virus infection in this cohort. This differs from previous studies that reported strong correlations between serum H1-specific IgG and a reduced risk of influenza A H1N1 virus infection.^40^^,^^41^ However, these studies were conducted in cohorts with limited vaccine exposure, whereas most of the participants in our cohort had received the seasonal tetravalent influenza virus vaccine. In addition, sampling occurred during a period when influenza virus circulation had been markedly reduced in the preceding years due to the COVID-19 pandemic, limiting mucosal exposure. High influenza virus-specific serum IgG in our cohort was therefore likely primarily generated by intramuscular vaccination rather than recent infection. In contrast, in cohorts with low vaccine coverage and a high viral spread, high serum IgG levels are more likely to reflect recent infection and may therefore coincide with a mucosal antibody response. In such settings, serum IgG may act as an indirect marker of mucosal immunity. Supporting this interpretation, we observed a modest but significant association between higher spike-specific serum IgG and protection against SARS-CoV-2 infection in our cohort, which may partly reflect recent infection and associated mucosal immunity. Taken together, we hypothesise that a vaccine-induced serum IgG response without a mucosal IgA response may not be enough to protect against infection. Recent SARS-CoV-2 studies support this interpretation by showing that nasal secretory IgA can exhibit greater neutralising potency and broader spike-binding breadth against Omicron subvariants than paired serum antibodies,^25^^,^^42^^,^^43^ while spike-specific mucosal IgA appears to correlate more strongly with protection against SARS-CoV-2 infection than spike-specific serum IgG.^7^^,^^25^ Indeed, in this cohort, regardless of the higher serum IgG levels, intramuscular tetravalent influenza and SARS-CoV-2 mRNA vaccinations were not associated with reduced infection risk, consistent with their limited induction of mucosal IgA. Historical data have indicated that the effectiveness of seasonal influenza virus vaccines in protection against infection has varied mainly based on how well the circulating strain matched the vaccine strain.^44^ National surveillance data confirm that the dominant circulating H1N1 strains in Sweden during the 2023–2024 winter season were well matched to the egg-adapted A/Victoria/4897/2022 strain, which was included in the 2023–2024 seasonal tetravalent inactivated split-virion influenza vaccine (Vaxigrip Tetra).^31^ Furthermore, the H1-specific antibodies analysed in this study were directed against A/Wisconsin/67/2022, which is also antigenically close to the aforementioned strains. The lack of association with protection against infection is therefore unlikely to be due to a mismatch between the circulating strain, the vaccine strain, or the antigen used for antibody analysis but could be explained by the absence of mucosal IgA induction from intramuscular vaccinations. Importantly, however, these vaccines are highly effective at preventing symptomatic and severe disease^45^^,^^46^ underscoring that protection against infection and protection against severe outcomes are distinct immunological endpoints.

The absence of a mucosal IgA induction or boost following intramuscularly delivered influenza virus and SARS-CoV-2 vaccination aligns with previous findings demonstrating that mucosal IgA responses following intramuscular inactivated influenza^5^^,^^13^ and SARS-CoV-2 vaccination are absent or very limited.^6^^,^^47^^,^^48^ The mucosal immune system consists of specialised lymphoid cell populations that differ from their systemic counterparts. To elicit strong mucosal antibody responses, antigen exposure at the mucosal surface is likely essential.^49^ A number of mucosal vaccines targeting SARS-CoV-2 have recently received approval for human use but clinical data are still scarce.^50^ Additionally, several mucosal vaccine candidates, targeting influenza virus or SARS-CoV-2 are currently under development.^51^, ^52^, ^53^ Preclinical data also highlight that mucosal antigen delivery induces local pathogen-specific IgA production in the respiratory mucosa,^54^, ^55^, ^56^, ^57^, ^58^ underscoring the importance of administration site to elicit mucosal IgA antibodies.

Although our findings are strengthened by the prospective screening design with active surveillance of influenza virus and SARS-CoV-2 infections enabling a test bed for correlates of protection against infection, several limitations should be considered. First, the relatively small number of influenza A H1N1-infected individuals during the qPCR screening period precluded the possibility of defining a protective mucosal IgA threshold. In addition, the low number of infections with other influenza virus subtypes limited subtype-specific analyses. Future multicenter studies with larger numbers of infection events will therefore be required to establish mucosal antibody thresholds and to evaluate protection against additional influenza virus subtypes. Second, functional assessment of mucosal antibodies would provide additional mechanistic insight. However, live-virus neutralisation assays in nasal secretions remain technically challenging due to limited sample volumes, low immunoglobulin concentrations, and matrix interference. Nonetheless, previous studies have demonstrated strong correlations between binding antibody titres and live virus microneutralisation for both ancestral and Omicron SARS-CoV-2 in blood, as well as strong correlations between serum binding antibody titres, microneutralization, and HAI for influenza virus in blood.^8^^,^^59^ Finally, we lacked detailed information on symptom severity and duration of the included infections and the use of personal protective equipment and exposure to infected household contacts during follow-up, and these factors could therefore not be accounted for in the analyses.

The COVID-19 pandemic has highlighted our vulnerability to emerging respiratory pathogens. Effective prevention of respiratory virus infection and transmission remains an ongoing challenge, despite the important role of existing vaccines in reducing severe illness. Despite major advances in vaccine development, current strategies largely focus on inducing systemic immunity through intramuscular vaccination. While these vaccines are effective at reducing severe disease and mortality, which remains their central public health benefit, our results underscore their limited capacity to prevent infection, replication and subsequent viral spread, enabling the emergence and transmission of mutated variants. Our findings furthermore underscore the importance of antigen-specific mucosal IgA in protection against infection with influenza A virus and SARS-CoV-2, both of which entail a high pandemic potential. Strategies focusing on the generation of robust respiratory mucosal IgA responses are urgently needed to reduce infection rates and serve as a pandemic preparedness measure.

## Contributors

**Oscar Bladh:** Writing: original draft, Writing: review & editing, Conceptualisation, Study design, Methodology, Formal analysis, Investigation, Data curation, Visualisation. **Tamás Pongrácz:** Visualisation, Formal analysis. **Katherina Aguilera:** Investigation, Resources. **Matilda Berkell:** Methodology, Data curation. **Ulrika Marking:** Methodology. **Nina Greilert Norin:** Investigation, Resources. **Ali Rihani:** Methodology, Investigation. **Jessica Alm:** Supervision, Resources, Methodology. **Florian Krammer:** Writing: review & editing. **Mikael Åberg:** Writing: review & editing, Resources, Methodology, Data curation, Supervision. **Charlotte Thålin:** Writing: review & editing, Supervision, Resources, Project administration, Funding acquisition, Conceptualisation, Data verification. All authors critically revised the manuscript for important intellectual content and approved the final version submitted for publication. Charlotte Thålin (corresponding author) and Oscar Bladh accessed and verified the underlying data reported in this manuscript.

## Data sharing statement

De-identified individual participant data that underlie the results reported in this article (text, tables, figures, and appendices) will be made available to qualified researchers upon reasonable request to the corresponding author (C.T.), subject to approval by the institutional review board and a data use agreement. Data will be available for academic, non-commercial purposes, and requests will be considered for up to 10 years after publication. Supporting materials such as the study protocol and statistical code are available upon request.

## Declaration of interests

Florian Krammer declares the following conflicts of interest. The Icahn School of Medicine at Mount Sinai has filed patent applications regarding influenza virus vaccines on which FK is listed as inventor. The Icahn School of Medicine at Mount Sinai has filed patent applications relating to SARS-CoV-2 serological assays, NDV-based SARS-CoV-2 vaccines influenza virus vaccines and influenza virus therapeutics which list FK as co-inventor and FK has received royalty payments from some of these patents. Mount Sinai has spun out a company, Castlevax, to develop SARS-CoV-2 vaccines. FK is co-founder and scientific advisory board member of Castlevax. FK has consulted for Merck, GSK, Sanofi, Gritstone, Curevac, Seqirus and Pfizer and is currently consulting for 3rd Rock Ventures and Avimex. The Krammer laboratory is also collaborating with Dynavax on influenza vaccine development. The remaining authors have no conflicts of interest to declare.
