# Supplementary material for: The role of mucosal IgA in protection against influenza A H1N1 virus infection in a real-world setting
Source: eBioMedicine. 2026 Jun 30;129:106359. doi: 10.1016/j.ebiom.2026.106359 (PMC13342958; doi:10.1016/j.ebiom.2026.106359)
Supplement: Supplementary Material [file mmc1.pdf]

The role of mucosal IgA in protection against influenza A H1N1 virus infection in a real-world setting - Supplementary data

Table of contents

Methods 2
RNA Extraction and RT-qPCR Analysis 2
Supplementary Figure 1 3
Table S1 4
References 5

RNA Extraction and RT-qPCR Analysis

For influenza virus detection RNA was extracted from 180 µL of each sample using the MGI Viral DNA/RNA Extraction Kit (Cat: 1000020261, MGI Tech, China) on an automated magnetic bead–based platform. Extracted RNA was analyzed with in-house one-step RT-qPCR assays targeting pan-Influenza A (INFA), Influenza A H1N1, and human RNase P (internal control). Reactions were performed in duplicate using the Reliance One-Step Multiplex Supermix (Cat: 12010221, Bio-Rad, USA) with ROX (INFA), FAM (H1N1), and Cy5 (RNase P) reporter dyes on a Bio-Rad CFX384 system. Cycling conditions were 50 °C for 30 min, 95 °C for 1 min, followed by 44 cycles of 95 °C for 15 s and 60 °C for 30 s with fluorescence acquisition at 60 °C. Primers and probes for the in-house assays were synthesized by Integrated DNA Technologies (IDT, Coralville, IA, USA). Exact sequences are not disclosed due to ongoing IP protection efforts; however, contextual information is provided in accordance with MIQE (Minimum Information for Publication of Quantitative Real-Time PCR Experiments) guidelines [1]. The **H1N1 assay** targets the hemagglutinin (HA) segment 4 (KR611222.1), with an anchor nucleotide at position 101 and a context sequence length of 239 bp. The **pan-Influenza A assay** targets the matrix protein (segment 7; PP576969.1), with an anchor nucleotide at position 40 and a context sequence length of 178 bp. The **human RNase P assay** targets NM_001104546.2, with an anchor nucleotide at position 39 and a context sequence length of 88 bp. Melting temperatures (Tm) and GC content for the primers and probes were designed to optimize amplification efficiency and specificity. The anchor nucleotide is defined as a nucleotide contained anywhere within the probe sequence. The context sequence length represents the amplicon plus surrounding bases as recommended by MIQE guidelines for primer/probe disclosure. Positive control of H1N1 consisted of genomic RNA of Influenza A H1N1 virus (ATCC VR-1520; American Type Culture Collection, Manassas, VA, USA). In parallel, SARS-CoV-2 RT-qPCR was performed in duplicate using the Sansure IVD one-step RT-qPCR kit (Cat: S3102E, Sansure Biotech, China), targeting ORF1ab and N genes with human RNase P as an internal control. Reactions were run in 96-well plates on a CFX96 real-time PCR system (RRID:SCR_018064, Bio-Rad Laboratories, Hercules, CA, USA) as previously described (Hober A et al 2021 PMID: 34747696, Marking et al 2023 PMID 36949041). Each plate included a no-template control, a positive control from the kit, and two extraction blanks. Data were analyzed using CFX Maestro software (Bio-Rad, USA).

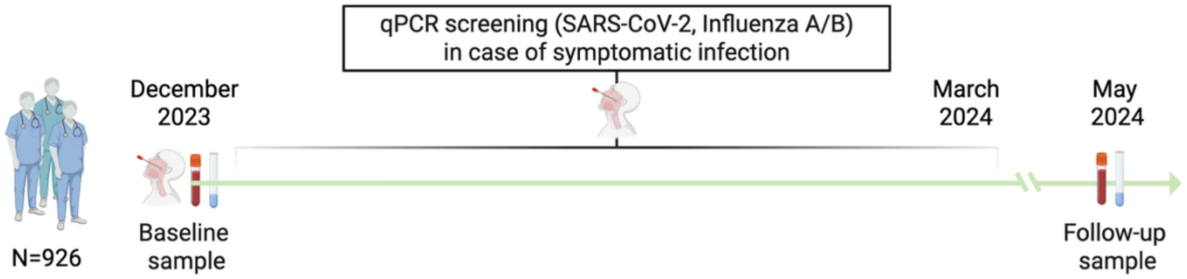

Figure S1. Study design and qPCR screening timeline.

Schematic overview of the study design. In December 2023, 1049 healthcare workers were sampled for baseline nasal IgA and serum IgG levels of whom 926 were enrolled in a 4-month home-based qPCR screening program for symptomatic SARS-CoV-2 and influenza A/B infections. Self-administered nasal/oropharyngeal/saliva swabs were collected at symptom onset and analyzed by qPCR. Follow-up sampling post infection was performed in May 2024.

Table S1 - Incidence rate ratios for influenza A H1N1 virus, and SARS-CoV-2 infection

Variable	Unadjusted IRR (95% CI)	p	Adjusted IRR (95% CI)	p
**Influenza A H1N1 virus infection - baseline antibody levels above median**				
Nasal H1-specific IgA above median	0.27 (0.06-0.87)	0.046	0.23 (0.05-0.75)	0.026
Serum H1-specific IgG above median	1.49 (0.54-4.45)	0.449	2.03 (0.70-6.62)	0.206
Age at baseline	-	-	1.05 (1.00-1.11)	0.070
Sex (male vs female)‡	-	-	-	-
**Influenza A H1N1 virus infection - per duplication of antibody levels†**				
Nasal H1-specific IgA	0.74 (0.55-0.97)	0.033	0.69 (0.51-0.92)	0.014
Serum H1-specific IgG	1.02 (0.70-1.54)	0.934	1.15 (0.76-1.83)	0.556
Age at baseline	-	-	1.06 (1.00-1.13)	0.048
Sex (male vs female)‡	-	-	-	-
**SARS-CoV-2 infection - baseline antibody levels above median**				
Nasal spike-specific IgA above median	0.29 (0.15-0.53)	<0.001	0.33 (0.17-0.60)	<0.001
Serum spike-specific IgG above median	0.40 (0.22-0.69)	0.001	0.46 (0.25-0.83)	0.012
Age at baseline	-	-	1.00 (0.97-1.02)	0.895
Sex (male vs female)	-	-	1.69 (0.77-3.31)	0.153
**SARS-CoV-2 infection - per duplication of antibody levels†**				
Nasal spike-specific IgA	0.74 (0.66-0.82)	<0.001	0.75 (0.67-0.84)	<0.001
Serum spike-specific IgG	0.86 (0.78-0.99)	0.019	0.93 (0.81-1.10)	0.342
Age at baseline	-	-	0.99 (0.97-1.02)	0.489
Sex (male vs female)	-	-	1.69 (0.77-3.32)	0.155
**SARS-CoV-2 vaccinated vs non-vaccinated**				
Vaccinated (ref: non-vaccinated)	0.66 (0.25-1.43)	0.340	0.69 (0.26-1.52)	0.398
Age at baseline	-	-	1.00 (0.97-1.02)	0.741
Sex (male vs female)	-	-	1.58 (0.72-3.10)	0.215
**Influenza vaccinated vs non-vaccinated**				
Vaccinated (ref: non-vaccinated)	1.19 (0.43-3.40)	0.734	1.06 (0.38-3.03)	0.917
Age at baseline	-	-	1.05 (0.99-1.11)	0.104
Sex (male vs female)	-	-	0.56 (0.03-2.77)	0.572

IRR = incidence rate ratio; CI = confidence interval. Unadjusted IRR from separate univariable Poisson regression for each primary exposure. Adjusted IRR from multivariable Poisson regression including all listed variables. All models include log(time at risk) as offset. Age and sex are included as covariates only; univariable IRR were not calculated for these variables (-). For vaccine effectiveness analyses, vaccinated participants were defined as those vaccinated within 30 days prior to baseline. Participants vaccinated 31-180 days prior to baseline were excluded. † Restricted to participants with antibody levels above the assay cut-off. ‡ Sex was omitted from influenza A H1N1 virus infection models because only one infected participant was male, precluding stable estimation of sex as a covariate.

References

1. Bustin SA, Benes V, Garson JA, et al. Primer Sequence Disclosure: A Clarification of the MIQE Guidelines. Clinical Chemistry 2011;**57**(6):919-21 doi: 10.1373/clinchem.2011.162958.
